# Superficial Surgical Site Infections in Primary Total Joint Arthroplasty: A Retrospective Analysis of Topical Anti-Biofilm Therapy

**DOI:** 10.7759/cureus.39490

**Published:** 2023-05-25

**Authors:** Joseph P Kelly, Andrew S Bae, Jacob Taunton, Achraf Jardaly, Robert M Harris

**Affiliations:** 1 Orthopaedic Surgery, The Hughston Foundation, Columbus, USA; 2 Orthopaedic Surgery, The Hughston Clinic, Columbus, USA; 3 Orthopaedic Surgery, Jack Hughston Memorial Hospital, Phenix City, USA; 4 Trauma and Orthopaedics, The Hughston Clinic, Columbus, USA

**Keywords:** infected hip and knee arthroplasty, post-op complications, biofilm eradication, orthopedics infections, biofilm effective antimicrobial, total joint arthroplasty

## Abstract

Introduction

Surgical site infections (SSI) following orthopedic procedures can cause significant morbidity and mortality, particularly in total joint arthroplasty. Biofilm formation in surgical wounds has made it difficult to prevent and treat these infections. SURGX® Antimicrobial Wound Gel (Next Science, Jacksonville, Florida, USA) was developed to disrupt biofilm formation but has not been evaluated in prophylactic use in total joint arthroplasty to prevent superficial SSI.

Methods

A retrospective chart review was performed at a single institution comparing the rate of SSI in patients undergoing primary total hip arthroplasty (THA) and total knee arthroplasty (TKA). SSI data were collected from patients with standard postoperative dressings (Group A: Control) and patients with SURGX® applied as part of a standardized dressing following THA/TKA (Group B: Study). Rates of SSI were compared.

Results

SURGX® was administered to 120 patients, including 91 TKAs and 29 THAs. The overall infection rate in this cohort was 2.5%. No superficial site infections developed. The control group constituted 566 patients, with 386 TKAs and 180 THAs. The infection rate was 1.24%, which included one superficial infection. Binary logistic regression did not show different odds of developing infections with the use of SURGX® (OR = 2.23, 95% CI: 0.54-9.13, p* = *0.27).

Conclusion

In our small retrospective study, Next Science SURGX® Antimicrobial Wound Gel did not demonstrate a statistically significant difference in the rate of superficial SSI in total joint arthroplasty; however, Group B did not have any superficial SSI.

## Introduction

Surgical site infections (SSIs) may be associated with significant morbidity and mortality, which is burdensome to both patients and the healthcare system. SSI in orthopedic procedures not only increases healthcare costs by 300%, but patients also have substantially greater physical limitations and reduced quality of life [1]. In some facilities, orthopedic surgery accounts for significant rates of SSI [2]. Several risk factors for SSI are modifiable and include host factors such as age, sex, and medical co-morbidities [3,4]. SSIs are potentially preventable complications and a great amount of time and resources have been dedicated to protocols and products that aim to reduce post-operative SSI rates. SSI can range from superficial infections involving the skin and/or subcutaneous tissues or deeper tissues, organs, or implants, leading to more serious complications [2]. The bacterial load present in the wound may be the most important factor attributing to the frequency and severity of SSI. Biofilm formation in the continuum of bacterial colonization of wounds complicates the issue of SSI. The CDC has reported that over 65% of hospital-acquired infections can be attributed to biofilm formation [5]. Biofilms are complex microenvironments that have proven extremely difficult to eradicate. Extracellular polymeric substance accounts for 80% of biofilm volume. Its chemical composition contributes to the properties of the biofilm itself and, therefore, often contributes to its antimicrobial resistance [6,5,7]. In the setting of orthopedic surgery, biofilm-forming bacterial strains have been found to be present more often in non-fluid tissues, particularly bone and surrounding soft tissues [8]. Management of biofilms has understandably become a major point of interest in infection management in recent years. The first step in mitigating biofilms should involve strategies to specifically prevent their formation [5]. Various methods have been developed to prevent biofilm formation. The pH of wounds has been consistently associated with the ability of bacterial quorum sensing and biofilm formation [9]. Surfactant-based dressings have been shown in animal models to effectively eliminate established biofilms when combined with minor debridement techniques [10]. Next Science (Jacksonville, Florida, USA) Wound Gel technology (SURGX®) utilizes a pH buffer system and benzalkonium chloride buffer system to chelate calcium and remove bacterial membrane proteins, and destabilize biofilm matrices [11]. SURGX® is an FDA-cleared product that has been shown in animal models to prevent biofilm development of both Gram-positive and Gram-negative pathogens in fresh wounds. In addition, it can also eliminate bacteria and establish biofilms from already-infected wounds without apparent risk of the formation of resistant colonies [12]. Wolcott R previously demonstrated that adding anti-biofilm wound gel to topical antimicrobials significantly increased wound healing [13]. The efficacy of such products was further established by Kim D et al., who demonstrated increased wound healing when combined with debridement when compared to debridement alone [11]. Anti-biofilm therapies such as Next Science’s SURGX® Antimicrobial Wound Gel have not been evaluated in the context of acute surgical incisions to prevent post-operative SSI. In this study, we investigate the efficacy of this product in preventing superficial post-operative infections in primary total knee and hip arthroplasty (TKA and THA). 

## Materials and methods

We performed a retrospective cohort study of patients having undergone primary THA or TKA at Jack Hughston Memorial Hospital (JHMH) (Phenix City, Alabama, USA) between March 10, 2020 and July 10, 2020 (Group B: Study). This was cross-referenced with the list of documented patients who had the SURGX® product applied after closure as part of their surgical dressing. The product was applied to the skin following closure prior to the application of a standard dressing. Six surgeons were identified as having used the product at the completion of their cases in this time frame. SSIs were recorded by the JHMH manager for infection control through surveillance of admissions to the same facility and monthly surveys by the surgeons. For the purpose of this study, superficial SSIs were defined as infections involving only the skin and subcutaneous tissue. Deep SSIs were defined as infections involving deep tissues, such as fascia and muscle layers. Organ space SSIs were defined as infections involving any part of the anatomy that had been opened or manipulated during an operation. SSI data was recorded in the same timeframe as above. SSI data of the same six surgeons from March 10, 2019 to July 10, 2019, were obtained for use as a control (Group A: Control). Charts were reviewed for resultant treatment for SSI, and operative notes were reviewed for details regarding the extent of infection for any patient treated surgically. Superficial, deep, and organ level (involving implants or osseous structures) were all recorded. Primary arthroplasty procedures were included due to the documented increased risk of post-operative infection in the revision setting. Only acute infections within 30 days of the procedure were included. This study protocol was approved by The Hughston Sports Medicine Center Institutional Review Board (Columbus, Georgia, USA; approval #HIRB2020-08).

## Results

This study included 686 patients in both Group B: Study (n = 120) and Group A: Control (n = 566). The average age was 65.7 years, and there was a slight female predominance (n = 369, 53.8%). Patient demographics are presented in Table 1. There were no demographic differences in patients between both groups.

**Table 1:** Patient demographics.

	Total (n = 686)	SURGX (n = 120)	Control (n = 566)	P-value
Age	65.7 ± 10.5	65.6 ± 10.8	65.7 ± 10.5	0.92
Sex Female Male	369 (53.8%) 317 (46.2%)	63 (52.5%) 57 (47.5%)	306 (54.1%) 260 (45.9%)	0.76
BMI	33.3 ± 7.1	33.5 ± 7.4	33.3 ± 7.0	0.75
Laterality Left Right	328 (47.8%) 358 (52.2%)	53 (44.2%) 57 (55.8%)	275 (48.6%) 291 (51.4%)	0.38
Smoking	117 (17.1%)	14 (11.7%)	103 (18.2%)	0.20
Diabetes	143 (20.8%)	30 (25.0%)	113 (20.0%)	0.43

In Group B: Study, SURGX® was administered to 120 patients, including 91 primary TKAs and 29 primary THAs. The overall infection rate in this cohort was 2.5%; however, this included two organ space infections and one deep infection. No superficial site infections were reported. Furthermore, no adverse events such as skin irritation or allergy were encountered in this group. Group A was the control group of 566 patients, with 386 primary TKAs and 180 primary THAs. The infection rate in this cohort was 1.24%. This included one superficial infection, three organ space infections, and three deep infections. Binary logistic regression was performed and did not show a difference in odds of developing infections with the use of SURGX® (OR = 2.23, 95% CI = 0.54-9.13; p = 0.27). Infection data are presented in Table 2.

**Table 2:** Rates of infection.

Type		SURGX			Control			Total
Location		Knee	Hip	Total	Knee	Hip	Total	
Infection Rate	Superficial	0	0	0	0	0.56%	0.18%	0.15%
	Organ Space	2.20%	0	1.67%	0.26%	1.11%	0.53%	0.73%
	Deep	0	3.45%	0.83%	0.26%	1.11%	0.53%	0.58%
	Total	2.20%	3.45%	2.50%	0.52%	0.73%	1.24%	1.46

## Discussion

Post-operative SSI remains a major complication following total joint arthroplasty surgery. Post-operative infections, as a whole, are devastating to both the patient and the healthcare system. With the number of total joint arthroplasty procedures continuing to increase from year to year and the emergence of drug-resistant pathogens, significant resources continue to be invested in research and protocols to attempt to decrease post-op infection rates. Biofilms have become an increasing focus of research surrounding SSI in recent years. Biofilm formation significantly increases the ability of infectious organisms to evade the immune response and procure relative resistance to common antimicrobial therapies, making treatment of both superficial and deep infections extremely difficult. New products such as Next Science's SURGX® Wound Gel have been developed to destroy biofilm and prevent its ability to form. It is an antimicrobial gel that is applied to the wound's surface and attacks the biofilm molecular structure. Next Science SURGX® Wound Gel disrupts the biofilm's extracellular polymeric substances (EPS) by interrupting the bonds separating the EPS into individual polymers, thus exposing the vulnerable bacteria [14]. Although this product has been previously shown to be effective in both animal studies and chronic wounds, its efficacy has not been studied in terms of prophylactic use in elective surgery in preventing superficial SSIs. The results of our limited retrospective study did not demonstrate a statistically significant difference in superficial SSI between standard dressing and Next Science SURGX® Wound Gel with standard dressing in primary hip and knee arthroplasty. The wound gel is applied over the closed incision as part of the final surgical dressing, which may explain the difference between the superficial vs. deep vs. organ SSI rates. Other possible factors contributing to our findings are the length of hospital stay, patient co-morbidities, and surgeon operative technique and surgery [15]. However, there were a comparatively low number of TJAs performed overall in 2020 due to COVID-19 restrictions in elective surgery at the facility. There were no superficial SSIs in Group B-Study patients.

Our retrospective study does have several limitations. The single-center nature of this study does not allow for the capture of patients in either cohort who may have been evaluated and treated for SSI at a different facility. The three-month study timeframe is also limited, especially in the context of SSI, as this would not capture patients who develop more indolent or slow-progressing infections. Although there is a standardized protocol for prepping and draping, antibiotic administration, and operative infection control at JHMH for total joint arthroplasty procedures, the inclusion of multiple (six) surgeons is a potential confounding variable that may influence the SSI rate. There was also a significant decrease in the number of cases performed in 2020 due to restrictions enacted during the COVID-19 pandemic affecting the potential outcomes; however, the SSI rate for both Group A and B was comparable with previously reported national SSI data. There are opportunities for further study. Although our study did not identify any statistically significant results in preventing superficial infections, a larger, multicenter, prospective study would be useful to explore the possible correlation between SURGX® and its efficacy in decreasing superficial infections. Further studies should also include longer follow-ups to more adequately capture post-operative infections.

## Conclusions

Next Science SURGX® Wound Gel did not demonstrate a statistically significant effect on the rate of superficial SSI in total joint arthroplasty. The use of it did result in an overall decrease; however, larger prospective, randomized studies with significantly more patients could be beneficial in further establishing any potential advantage to its use in reducing post-operative infection rates.
